# Takotsubo Cardiomyopathy Following the Resection of a Cerebellopontine Angle Meningioma

**DOI:** 10.7759/cureus.95298

**Published:** 2025-10-24

**Authors:** Mohammed Sidayne, Jamal Ouachaou, Driouich Aicha, Fatimazahrae El khettab, Zarrouki Youssef

**Affiliations:** 1 Department of Anesthesia and Critical Care, Mohammed VI University Hospital of Marrakech, Marrakech, MAR

**Keywords:** cardiovascular, general physiology, intensive care, neuroanesthesia, stress

## Abstract

Takotsubo cardiomyopathy is a rare, reversible form of acute left ventricular dysfunction triggered by a catecholamine surge in response to severe stress. We report the case of a 42-year-old woman who developed acute ECG changes, regional wall motion abnormalities with apical ballooning, and markedly elevated cardiac biomarkers 72 hours after resection of a cerebellopontine angle meningioma. Coronary angiography excluded obstructive coronary disease, and supportive management led to rapid recovery of ventricular function. This case highlights the need for early recognition of perioperative Takotsubo cardiomyopathy in neurosurgical patients because its clinical presentation can mimic acute coronary syndrome and because misdiagnosis may lead to inappropriate antithrombotic management. We recommend prompt echocardiography and coronary angiography when clinically feasible, multidisciplinary discussion, and tailored hemodynamic support in suspected cases.

## Introduction

Takotsubo cardiomyopathy (TCM), also known as stress-induced cardiomyopathy or “broken heart syndrome,” is an acute but reversible form of left ventricular dysfunction that occurs in the absence of obstructive coronary artery disease [1]. It is typically triggered by intense physical or emotional stress, with the underlying mechanism involving an exaggerated sympathetic response leading to catecholamine-induced microvascular spasm, myocardial stunning, and transient systolic dysfunction [2,3]. The most widely used diagnostic criteria are those proposed by the Mayo Clinic (2008), which define TCM by the presence of transient akinesia or dyskinesia of the apical and/or mid-ventricular segments of the left ventricle (classically manifesting as apical ballooning), the absence of angiographic evidence of obstructive coronary artery disease or acute plaque rupture, new ECG abnormalities such as ST-segment elevation or T-wave inversion, and the exclusion of other causes such as intracranial hemorrhage, pheochromocytoma, or myocarditis [4,5]. Another diagnostic tool, the InterTAK score, estimates the likelihood of TCM based on several clinical features, including female sex, psychiatric disorders, emotional or physical stress, absence of ST-segment depression, and prolonged QTc interval, with a score above 70 indicating a very high probability [6]. Perioperative TCM has been described across various surgical specialties, including general surgery, orthopedics, obstetrics, and neurosurgery, but remains rare and usually reported as isolated case reports [1,7]. Within neurosurgery, several cases have been documented, including those following pituitary adenoma resection [8] and fourth ventricle tumor surgery [1]. Here, we report a rare case of postoperative hemodynamic instability following cerebellopontine angle meningioma resection that progressed into stress-induced TCM, and we review the relevant perioperative literature, highlighting the importance of recognizing TCM in the neuroanesthesia and intensive care settings.

## Case presentation

A 39-year-old woman with no significant medical history was admitted for the surgical excision of a left cerebellopontine angle meningioma. She presented with incomplete signs of intracranial hypertension, vertigo, and progressive left-sided hearing loss over one year. Preoperative MRI revealed a lobulated extra-axial mass in the left cerebellopontine angle measuring 35 × 26 × 46 mm.

The tumor was resected via a retrosigmoid approach with complete microsurgical excision. No intraoperative blood transfusion or vasoactive support was required. Postoperatively, the patient was transferred after intubation to the surgical intensive care unit (ICU), ventilated without sedation, and she presented with miotic pupils. The initial postoperative period was marked by delayed awakening and minimal responsiveness. MRI performed at 48 hours revealed a small residual tumor compressing the trigeminal nerve and interruption of venous flow in the left jugular sinus, suggestive of thrombophlebitis. Anticoagulation therapy was discussed in a multidisciplinary team meeting.

**Figure 1:**
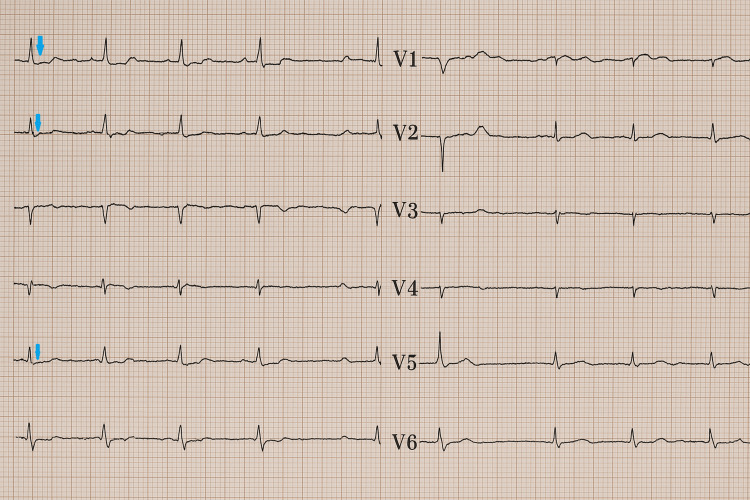
Postoperative ECG showing marked ST-segment depression (arrows mark areas of ST-segment depression)

Seventy-two hours after surgery, the patient developed acute respiratory distress with poor ventilatory synchronization, muscle fatigue, and desaturation. She subsequently exhibited marked hemodynamic instability with tachycardia and hypotension, poorly responsive to norepinephrine. Further investigations revealed significant ECG changes, including ST-segment depression and T-wave inversion in the inferolateral leads (Figure 1).

**Figure 2:**
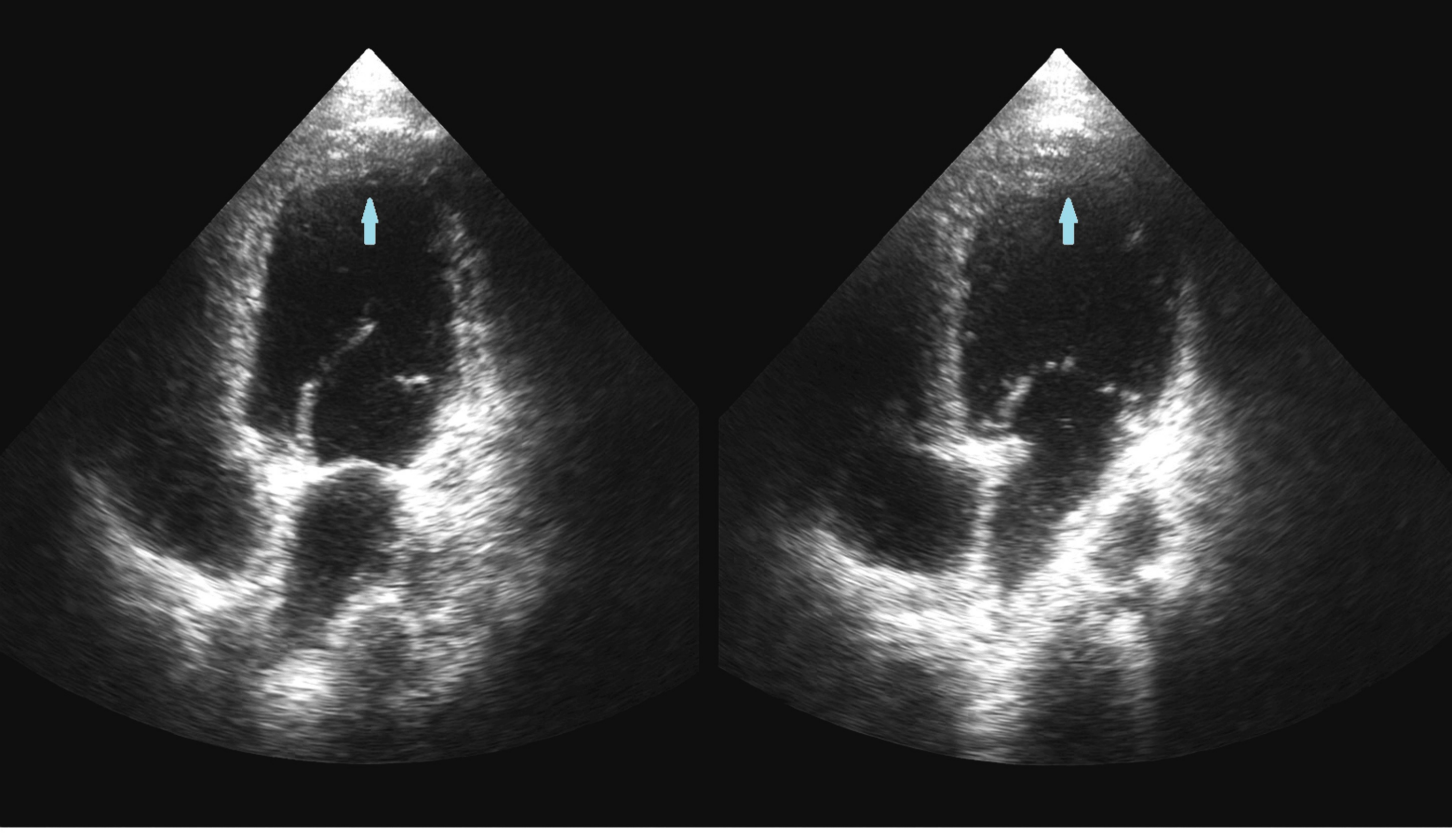
Transthoracic echocardiography (apical four-chamber view) showing apical ballooning (arrow)

Transthoracic echocardiography demonstrated mid-apical, inferoseptal, and inferior wall hypokinesia with characteristic apical ballooning (Figure 2).

Laboratory tests showed elevated troponin (414 ng/L) and N-terminal pro-B-type natriuretic peptide (NT-pro-BNP; 3,431 pg/mL) levels, while coronary angiography ruled out obstructive lesions. Laboratory reference ranges are assay-dependent. For example, the 99th percentile cutoff for high-sensitivity troponin T is ≈14 ng/L; commonly used age-adjusted NT-proBNP thresholds are ≈450 pg/mL (<50 years), ≈900 pg/mL (50-75 years), and ≈1800 pg/mL (>75 years). Hence, the patient’s troponin (414 ng/L) and NT-proBNP (3,431 pg/mL) levels were classified as being markedly elevated.

Based on these findings, a diagnosis of TCM was established. Management included bisoprolol (2.5 mg), dobutamine infusion, and supportive sedation. The patient’s hemodynamic status and left ventricular function improved rapidly, with normalization of echocardiographic findings within 48 hours. Due to persistent ventilatory difficulties and a poor cough reflex, a tracheostomy was performed on postoperative day seven. Repeat echocardiography on day 10 confirmed the complete recovery of left ventricular function with no regional wall-motion abnormalities. By postoperative day 12, the patient was fully awake, hemodynamically stable, and successfully transferred back to the neurosurgery ward.

## Discussion

Hemodynamic instability is a common intraoperative and postoperative event during posterior fossa surgery, occurring in up to 56% of brainstem tumor resections [7,9]. This may result from direct manipulation of cranial nerve nuclei or indirect stimulation of the dorsal medulla [10]. Reports of TCM following intracranial surgery are extremely rare, with only a few prior cases described in the literature. Our case appears to be only the second involving a cerebellopontine angle tumor.

The estimated prevalence of TCM is 5.2 per 100,000 in women and 0.6 per 100,000 in men, with approximately 30% of cases associated with surgery or hospitalization. The primary pathophysiological mechanism is a catecholamine surge triggered by acute stress. In the perioperative setting, this can result from preoperative anxiety, inadequate anesthesia or analgesia, or surgical manipulation near autonomic centers, as in our case [7].

Clinically, TCM often mimics acute coronary syndrome (ACS), presenting with ECG abnormalities, elevated cardiac biomarkers, hypotension, or pulmonary edema. However, in sedated or critically ill patients, the classic symptoms of chest pain or dyspnea may be absent [5]. Prompt coronary angiography is crucial to differentiate TCM from ACS, especially in neurosurgical patients for whom anticoagulation or antiplatelet therapy could pose life-threatening risks [5]. Management is primarily supportive, including diuretics, beta-blockers, angiotensin-converting enzyme (ACE) inhibitors, and careful hemodynamic monitoring. In severe cases, mechanical circulatory support (intra-aortic balloon pump, extracorporeal membrane oxygenation, or ventricular assist devices) may be required. Non-catecholaminergic inotropes such as milrinone or levosimendan can be used in cases of cardiogenic shock [3,5,7,11].

Although most patients recover normal left ventricular function within four weeks, short-term complications such as arrhythmias, stroke, or cardiogenic shock may occur, with mortality rates comparable to those of acute myocardial infarction. Recurrence, though rare, is possible, particularly in patients undergoing multiple surgeries under general anesthesia [12]. Early recognition, multidisciplinary discussion, and hemodynamic optimization are key to improving outcomes in this patient population.

## Conclusions

TCM is an uncommon but important cause of acute perioperative hemodynamic deterioration that can closely mimic ACS. In neurosurgical patients, particularly after posterior fossa or brainstem procedures, autonomic disturbances and catecholamine surges may precipitate TCM. Therefore, a high index of suspicion is warranted when new ECG changes, elevated cardiac biomarkers, and left ventricular dysfunction are observed.

Early echocardiography and, when feasible, coronary angiography are essential to differentiate TCM from obstructive coronary disease and to avoid inappropriate antithrombotic therapy that could be harmful in neurosurgical patients. Multidisciplinary management, involving neuroanesthesia, cardiology, and intensive care teams, and individualized hemodynamic support can lead to rapid recovery of ventricular function, as demonstrated by our case. Increased awareness and prompt diagnostic workup should be emphasized in perioperative protocols to improve detection and outcomes of TCM in surgical populations.
